# Tunnel enabled programmable switches obfuscate network topology to defend against link flooding reconnaissance in software defined networking

**DOI:** 10.1038/s41598-025-19566-7

**Published:** 2025-10-10

**Authors:** Xiang Li, Jungmin Lee, Junggab Son, Yeonjoon Lee

**Affiliations:** 1 Department of Computer Science and Engineering, Major in Bio Artificial Intelligence, Hanyang University, Ansan 15588, South Korea; 2 Department of Computer Science, University of Nevada, Las Vegas, NV 89154 USA; 3 College of Computing, Hanyang University, Ansan 15588, South Korea

**Keywords:** Link flooding attacks, Programming protocol-independent packet processors, Software-defined networking, Programmable switches, Proactive defenses, Tunnel, Engineering, Electrical and electronic engineering

## Abstract

Recently, Software-Defined Networking (SDN) has emerged as an increasingly popular network paradigm due to its virtualization capabilities and flexibility. However, its robustness in link connectivity is threatened by Link Flooding Attacks (LFAs). To launch LFAs, adversaries use probing tools to infer network topologies and identify target links with bottlenecks. Thus, protecting SDN topologies against disclosure is crucial to ensure system security and preserve infrastructure functionality. We propose TEPS (Tunnel-Enabled Programmable Switches), a proactive defense system that dynamically obfuscates network topologies to defend against adversarial reconnaissance in SDN. TEPS generates false topologies by leveraging the flexibility of emerging programmable switches to construct customized tunnels and manipulate probing packets using the P4 language. This prevents adversaries from obtaining accurate knowledge of network topologies, making it difficult to reconstruct the true topologies. Furthermore, TEPS counters Round-Trip Time (RTT)-based fingerprinting attacks by dynamically adjusting packet delays and routing traffic to conceal RTT variations. Our evaluation demonstrates that TEPS effectively reduces the distribution of link importance in network topologies compared to the latest proactive defense method, thereby concealing bottlenecks and disrupting adversarial topology reconnaissance, including thwarting RTT-based fingerprinting attempts. Furthermore, by leveraging the capabilities of P4 switches, TEPS introduces minimal network overhead, with at most a 3% reduction in throughput and a 9.57% increase in resource utilization, showing practical feasibility under real-world operational constraints. By implementing TEPS, network administrators can enhance the security of their SDN infrastructures against LFAs and maintain robust connectivity through a lightweight approach.

## Introduction

Software-Defined Networking (SDN) has emerged as a transformative approach to modern networking due to its ability to address scalability, flexibility, and management challenges. By abstracting and separating the control and data planes, SDN provides flexible programmability over network protocols and topologies while simplifying network management. As a result, SDN is establishing itself as the next-generation networking paradigm ^1^. Protecting the network topology structure from disclosure is essential in SDN to prevent security breaches and unauthorized access to network infrastructure, ensuring that only authorized entities can access critical details. This strengthens resilience and improves the ability to effectively manage and control network resources in SDN.

However, SDN faces the threat of Link Flooding Attacks (LFAs)^2–4^, a new type of distributed denial-of-service (DDoS) attack. Unlike conventional DDoS attacks, LFAs aim to overwhelm the bandwidth of critical network links, potentially causing large-scale network paralysis, and leverage legitimate low-rate traffic that is difficult to distinguish from benign traffic. Adversaries first attempt to identify the network topology, often using path tracing and packet capture tools like Traceroute^5^to implement a successful LFA. They gather information on previously unknown topologies and analyze flow density by observing Round-Trip Time (RTT) through this probing process, allowing them to identify potential bottlenecks that could become target links. To disrupt the reconnaissance of the topology of LFAs in the SDN environment, a wide range of techniques have been proposed, including reactive defenses^6–13^and proactive defenses^14–25^. Although reactive defenses are activated only after an attack occurs, proactive defenses are designed to prevent attacks before they cause significant damage. These defenses take preemptive actions to increase the difficulty and cost of carrying out an attack, making it less likely that adversaries will attempt the attack in the first place and reducing the likelihood of successful attacks. Recent advancements in proactive defenses leverage machine learning for probing detection (e.g., ProTO^19^), dynamic topology obfuscation (e.g., NetObfu^20^, EqualNet^25^), and integrated deception frameworks (e.g., BottleNet^18^). These approaches adaptively disrupt adversarial reconnaissance by altering flow densities, mutating paths based on leakage thresholds, and combining multiple obfuscation techniques.

To achieve more effective proactive defense, we introduce the concept of tunneling. Tunneling is a crucial technique for secure transmission, often used to conceal information and protect privacy and anonymity. At its core, a tunnel encapsulates packets within new headers. Network forwarders process the encapsulated packets based solely on the instructions in the new headers without inspecting the original content. As a result, packets only need information about the entrance and exit points of the tunnel without requiring knowledge of the forwarding path (e.g., Google B4^26^, which leverages IPv4-in-IPv4 tunnels for cross-regional communication). This idea can also be applied to proactive defenses against topology reconnaissance, which relies on node responses along the path. By encapsulating probing traffic within a tunnel, the underlying physical links remain concealed, making it significantly more difficult to gather sufficient evidence to infer the entire topology. Furthermore, the emergence of programmable switches and protocol-independent switching architecture (PISA)^27^ has brought enhanced flexibility to SDN, enabling the application of tunneling techniques^28–39^. Network operators can customize processing pipelines and packet headers through Programming Protocol-independent Packet Processors (P4)^40^, a Domain-Specific Language (DSL) tailored for network programming. Building upon this foundation and the principles of tunneling theory, encapsulating probing traffic in the SDN data plane using a P4 program is feasible.

In this paper, we propose tunnel-enabled programming switches (TEPS), a proactive defense system designed to prevent network topology inference in SDN. TEPS encapsulates probing traffic directly in the data plane using custom special P4-programmed tunnels, which manipulate the header and forwarding behavior of probing packets to obfuscate reconnaissance efforts aimed at revealing the entire topology. Additionally, TEPS records probing packets as they enter and exit the tunnel to locate malicious bots and take further action to limit their behavior. Moreover, to counteract adversarial end-to-end topology inference, such as fingerprinting based on RTT or Network Tomography (NT)^41^, TEPS employs a mechanism that manipulates RTT in tunnel mode to increase the randomness of timing metrics. These mechanisms increase the complexity and difficulty of adversarial topology inference. Simulation results from several sets of real-world networks demonstrate that TEPS effectively defends against topological inference based on network tomography and prevents the leakage of topological information.

In summary, this paper makes contributions as follows. TEPS (Tunnel-Enabled Programmable Switches), a novel proactive topology obfuscation solution implemented on programmable switches, constructing a tunnel for probing packets using P4 language to protect SDN topologies from reconnaissance attacks is proposed. Experiments on the TEPS prototype are conducted using real network topologies, demonstrating its effectiveness in defending against reconnaissance attacks and its robustness against time-based fingerprinting. Furthermore, an in-depth analysis of existing proactive LFA defense methods is presented, along with a discussion of their potential improvements.

The organization of this paper is outlined below. The Background section provides background information, while the Related Work section surveys previous research on attacks targeting SDN and their countermeasures. The Methodology section presents a detailed explanation of the TEPS processing pipeline, and the Evaluation section shows the evaluation metrics and corresponding results. The Discussion section discusses the remaining issues, and we conclude the paper in the Conclusion and Future Work section. A complete list of abbreviations used in this paper is provided in Table 1.

**Table 1:** List of abbreviations.

**Abbreviation**	**Full Term**
SDN	Software-Defined Networking
LFA	Link Flooding Attack
DDoS	Distributed Denial-of-Service
RTT	Round-Trip Time
PISA	Protocol-Independent Switching Architecture
P4	Programming Protocol-Independent Packet Processors
DSL	Domain-Specific Language
TEPS	Tunnel-Enabled Programmable Switches
NT	Network Tomography
TTL	Time-to-Live
APR	Adversarial Path Reconnaissance
EL	Ensemble learning
HCF	Hop Count Filtering
LDOS	Low-rate Denial of Service
QoS	Quality of Service
IDP	Intelligent Data Plane
ML	Machine Learning
DNN	Deep Neural Network
ECMP	Equal-Cost Multi-Path
LISP	Locator/ID Separation Protocol
CRC	Cyclic Redundancy Check
GED	Graph Editing Distance

**Fig. 1:**
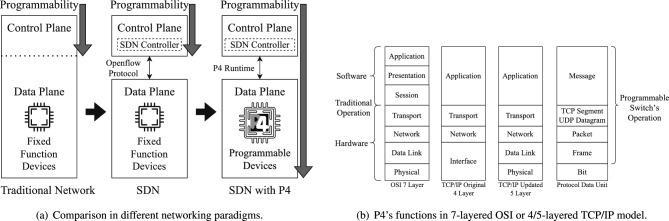
Evolution of P4-based SDN.

## Background

This section provides background information on concepts related to SDN and LFAs.

### PISA and P4

SDN abstracts physical devices as data planes and enables decision-making through a logically centralized virtual controller, which communicates with data planes via southbound APIs such as OpenFlow^42^ and P4Runtime^43^. With the emergence of PISA^27^, the development of OpenFlow slowed after its latest specification^44^. Enabled by the P4^40^ language, PISA provides an ideal architecture for autonomous processing pipelines in the data plane, eliminating the need for continuous controller involvement. PISA extends programmability to the data plane, allowing customization of packet headers and actions based on the P4 language standard^45^, thereby achieving protocol independence without relying on predefined protocol suites. Using inherent hardware advantages, PISA provides high-speed, line-rate performance while maintaining cost-effectiveness. Fig. 1 shows the evolution of SDN.

**Fig. 2:**
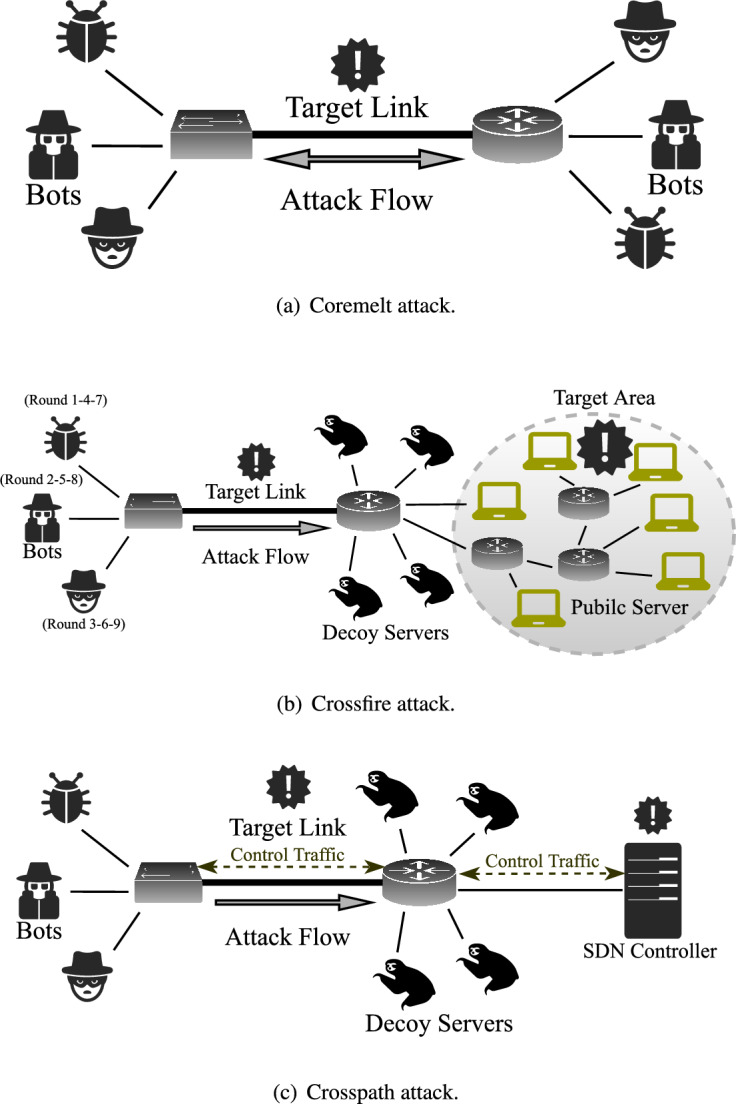
Simplified models of various types of LFAs.

### Time-to-Live and Traceroute

Traceroute, as strictly defined by IP and ICMP^46^, is crucial for measuring network performance. It relies on the TTL (Time-to-Live) field, sending a series of packets starting with a TTL values of 1, which increases by 1 with each subsequent packet. Routers along the path decrement the TTL value by 1, return their IP addresses and RTT, and forward the packet to the next hop. When the TTL value reaches zero, the router discards the packet and sends an *ICMP_time_exceeded* message (ICMP Type=11, Code=0). In traditional networks, the TTL decrement functionality is employed only by Layer 3 (Network Layer) devices, such as routers. Openflow-1.0 SDN^47^ initially lacked support for TTL decrement, but subsequent updates introduced this functionality, enabling Traceroute in OpenFlow. In addition, OpenFlow switches now support most of the functions of Layer 3 routers, which allows the possibility of launching Traceroute-based attacks in SDN.

### Link flooding attacks

**Fig. 3:**
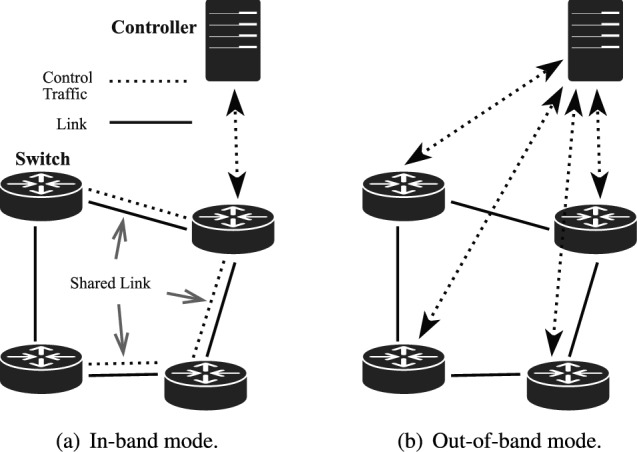
SDN control channel.

Coremelt^2^, Crossfire^3^, Crosspath^4^ are three different types of LFAs, as shown in Fig. 2. Coremelt and Crossfire are applicable in both traditional networks and SDN, while the Crosspath attack is specific to SDN environments with in-band control mode. Fig. 3a shows the in-band control mode, where the control channel shares the same physical or logical path as the data traffic in the data plane, resulting in *shared links*. In contrast, Fig. 3b shows the out-of-band control mode, where the control channel is separated from the data plane and carried over independent paths. Coremelt and Crossfire use universal tracing tools to identify target links with high traffic on the paths. In the Crosspath scenario, adversaries use Adversarial Path Reconnaissance (APR) to locate these *shard links* as targets. When installing reactive rules in OpenFlow-based SDN, the first packet of a flow is sent to the controller for rule inquiry and installation. Once the rule is installed, subsequent packets follow this rule until a hard timeout expires. Adversaries can measure the difference in RTT between the first two packets, both before and after a short burst of traffic. As the burst disrupts the flow, causing a noticeable variation in the RTT, a significant discrepancy between the two cases implies the presence of *shard links*.

## Related work

A wide range of methods have been proposed to counter LFA attacks, with some approaches using P4 programming in SDN environments.

### Centralized reactive defenses against LFAs

Centralized reactive approaches are activated once LFAs occur and rely on a central controller to send operational instructions to the data plane. SPIFFY^10^ proposed a temporary expansion of the bandwidth on the target link. They argued that bots would not increase their low rate to avoid detection, while benign hosts would increase their speed, making it possible to distinguish bots from legitimate hosts. Similarly, bots that do not adapt the routing are exposed through constant forced rerouting because the attack flows must remain at their original destination^11^. Among the several representative centralized reactive defenses^6–9,13^, CrossGuard is an OpenFlow-based SDN reactive defense designed to prevent control channel congestion. It prioritizes control traffic and limits the rate of attack data traffic to prevent bandwidth exhaustion. In addition, it employs an iterative dichotomy method to slice the mask and identify malicious traffic within subnets. ONSET^12^ uses optical topology programming to dynamically adjust the network topology and mitigate congestion.

### Proactive defenses against LFAs

Reactive defenses focus on addressing the flooding phase of LFA attacks, whereas proactive defenses aim to obstruct the probing phase by interfering with the adversary’s ability to locate network bottlenecks.

**Rerouting probing flows to avoid bottlenecks.** Linkbait^15^ introduces a set of unimportant links that act as bait, directing probing packets to these links and thus hiding the actual bottleneck link to avoid attacks. SDNHoneyNet^16^ deploys honey nodes and exposes a fake honey topology to adversaries, forcing traceroute flows with a TTL value of 1 to be directed to the honey topology. This concept is similar to the honeypot approach. Another approach^22,23^ involves adding a virtual cloud or overlay network to the physical topology. This virtual network expands the probing view, redirecting probing flows to a reflection topology connected by a GRE tunnel, which extends from actual nodes. Mirage^14^ interferes with APR to thwart cross-path attack by allocating alternate paths to control traffic. If no alternate paths are available, the short-lived rule treats the initial packets as new and sends them to the controller. The core concept behind this is that interference with the RTT calculation prevents adversaries from detecting shared links.

**Comprehensive topology deception.** NetHide^17^ computes a secure and usable virtual topology and deploys the obfuscated topology within the physical network. This is achieved by manipulating the TTL fields in traceroute packets through programmable switches to prevent probing flows from expiring or bypassing nodes, while also adding virtual links to ensure that probing results align with the virtual topology. ProTO^19^ employs machine learning algorithms to detect probing behavior, which is then followed by proactive obfuscation of the topology. The system delays all identified probing packets on the data plane via programmable P4 switches. NetObfu^20^ also calculates a secure virtual topology, causing different nodes to respond to packet probes and altering flow density. AntiTomo^21^ includes both a candidate forest generation algorithm and a network topology obfuscation algorithm. BottleNet^18^ is a comprehensive topology deception system that integrates various techniques such as redirecting probing flows, manipulating probing packet headers, and deploying virtual topologies. EqualNet^25^ continuously monitors the path-tracing flows and dynamically obfuscates the network whenever the level of topology leakage exceeds a specified threshold. This is done by generating path-tracing responses that include both real IP addresses of popular nodes and virtual IP addresses of ordinary nodes, making all links appear equal even when they are not. In addition, a comprehensive scheme is proposed that includes alternate routing for probing flows and random TTL decreases for probing packets^24^. These strategies prevent effective topology mapping and bandwidth throttling, mitigating bottleneck measurement.

### Limitations of existing works

Selective skipping of important nodes^17^ or random assignment of TTL decrements^24^ introduces the risk of being fingerprinted by adversarial network tomography, which is based on time metrics. These methods can reveal realistic timings and underlying physical paths, making it easier for adversaries to reconstruct the network topology. Such attacks leverage inconsistent or anomalous evidence from the RTT series table^48^, allowing adversaries to infer the topology despite obfuscation efforts.

### Miscellaneous cyber defenses via P4-based SDN

**DDoS mitigation.**

Poseidon^49^ and Jaqen^50^ provide network administrators with interactive, modular APIs, as well as high-level, developer-friendly policy expression primitives for forming strategies and runtime management. These systems instruct switches to execute different P4 programs based on the type of DDoS attack detected. SmartCookie^28^ integrates P4 switches with an agent server to monitor cookies and connection statuses within the data plane. It responds with a normal ACK to benign SYN requests while blocking malicious stream requests such as SYN flood attacks, one of the most prevalent DDoS attack.

**LFAs mitigation.**

Ripple^51^ and Mew^52^ leverage interactive APIs, high-level primitives, and runtime management. Ripple builds on existing OpenFlow-based SDN defenses and implements these on P4 switches, introducing new policies for enhanced protection. However, Mew implements distributed storage in the band for flows, significantly reducing the memory pressure on the switches and applying detection and mitigation modules to combat LFAs effectively. PLUTO^53^ leverages tree-based ensemble learning (EL) algorithms (XGBoost, RandomForest, LightGBM) to achieve robust Low-rate Denial of Service (LDoS) attack detection. EL models analyze time domain (e.g. TCP bandwidth degradation) features extracted from aggregate flow statistics. The choice of EL ensures high accuracy without feature normalization and aligns with P4’s match-action paradigm.

**Other attacks mitigation.** NETHCF^54^ filters spoofed IP traffic, a common method used in DDoS attacks, by implementing the Hop Count Filtering (HCF) scheme on programmable switches. It maintains an IP-to-Hop-Count mapping table that helps identify spoofed IP traffic by comparing the number of hops in the table with the hops in the incoming packets. P4Control^39^ provides policies against cross-host attacks, utilizing expressive network control primitives. These primitives allow the definition of defense policies that match cross-host flows, triggering appropriate counteractions to mitigate such attacks. Furthermore, SDN is also considered a key enabler for Quality of Service (QoS) and security in next-generation wireless sensor networks and is expected to create significant synergy when integrated with Machine Learning (ML) technologie^1,55^.

**Learning-based programmable switches.**

The integration of AI with P4-programmable switches has emerged as a paradigm to address real-time security challenges in SDN. NetBeacon^56^ pioneers an intelligent data plane (IDP) by embedding lightweight ML models (e.g., decision trees) directly into the programmable switches, It employs multi-phase sequential models to dynamically analyze flow-level features (e.g., packet size variance) and per-packet attributes, achieving line-speed traffic classification for multiple security policies (e.g., covert channel identification). By optimizing model representation via range marking, NetBeacon reduces hardware resource consumption while maintaining high accuracy. NetNN^57^ adopts deep neural networks (DNNs) in the data plane, eliminating manual feature engineering by processing raw packet bytes. It distributes DNN computations across switches via packet-carried intermediate results, achieving very high intrusion detection accuracy. Both works highlight the shift from control-plane-centric ML to data-plane-native inference, enabling microsecond-level response to attacks. PLUTO^53^ leverages EL. This encoding-based mapping converts tree paths into ternary matching tasks, enabling efficient in-network inference. EL’s ensemble approach enhances detection robustness, contributing to PLUTO’s superior performance in AUC, F1, and Recall metrics.

### Encapsulation in P4-based data plane

ONTAS^36^ enables the anonymization of packet fields, providing operators with a policy language that allows them to express anonymization tasks for specific fields. SPINE^37^ and PINOT^38^ encapsulate the packet header through an IPv6-in-IPv4 tunnel, preserving the original fields for information concealment. However, these solutions require encryption and decryption mechanisms in coordination with the controller. SmartCookie^28^ securely computes and verifies cookies using cryptographically robust hash functions to establish verified connections from end to end. Bhatnagar et al.^35^ implemented cryptographic measures to secure the SDN control channel. Although these measures ensure the integrity, authenticity and confidentiality of control traffic messages, control channels on shared links remain vulnerable to cross-path attacks^4^ and APR.

### Advanced technology

Olanrewaju et al.^58^ demonstrate how blockchain-based immutability can mitigate side-channel attacks in IoT mobility scenarios, providing valuable insights that directly inform TEPS’s topology obfuscation strategies against reconnaissance-driven link flooding attacks (LFAs). Adaptive routing models^59,60^ apply ant colony optimization for trust-based path selection on blockchain networks, offering SDN controllers a bio-inspired approach to dynamically manage encrypted tunnels. When integrated with P4’s match-action pipelines, these approaches enable real-time adjustment of obfuscation parameters in response to adversarial traffic analysis. Game-theoretic frameworks provide systematic methods for addressing adversarial behavior in programmable networks. For example, the Strategic Game Model (SGM)^61^ introduces formal techniques for detecting node misbehavior in IoT-cloud ecosystems, which could extend TEPS’s defense capabilities by modeling attacker-defender interactions through SDN telemetry. Likewise, multi-agent layered game formulations^62^ and collusion-resistant strategies ^63^ further enhance SDN’s ability to preempt multi-vector attacks—particularly relevant for P4-based data planes where low-latency responses to flow rule manipulation are critical.

## Methodology

In this section, we outline the threat model and detail the pipeline of TEPS, focusing on its operational algorithms.

### Design considerations and threat model

**Design considerations.** Existing topology obfuscation techniques with target certainty^17,18,20,21,25^ often rely on pre-made target effects with specific topologies. However, if adversaries are aware of the defensive goal (e.g., maximizing the difference), the original topology can be easily reversed. Thus, a defense designed solely to obstruct adversarial observation, without specific constraints for topological deception and lacking an explicit mapping between real and virtual topologies, would result in lower operational costs and higher robustness.

By manipulating the transmission path and the RTT values of the probing packets, the uncertainty in the timing-based metrics can be significantly increased. Our approach uses the concept of tunneling, which hides the underlying physical links. By encapsulating probing packets into tunnels, we can control the response of physical devices, increasing the difficulty of adversaries reconnaissance. This also complicates efforts for adversaries that rely on RTT-based NT, as they would need a comprehensive series of RTT across multiple hops to gather sufficient evidence.

Modern programmable switches with PISA^27^ and P4^40^, are suitable for: (1) the construction of customized packet headers and pipelines directly in the data plane, moving beyond the traditional OSI 7-layer model; (2) the guarantee of high line rate performance at Tbps speeds, regardless of the packet manipulation programs, significantly improving performance; (3) programmable switches are cost-efficient, providing comparable speed with lower cost and reduced power consumption. We construct a special tunnel on programmable switches under the instruction of the P4^45^ language.

**Threat model.** We assume that an attacker controls a set of bots capable of using Traceroute to locate all routers. By sending packets with incrementing TTL values from zero to the destination and collecting “timeout” information returned by nodes along the way, the attacker can map the forwarding path of packets. In the NT model based on RTT^41^, adversaries exploit the RTT measurements between various nodes to infer the underlying links. In addition, we assume that the attacker will not launch a blind attack that does not rely on topology mapping results.

### System overview and design

**Fig. 4:**
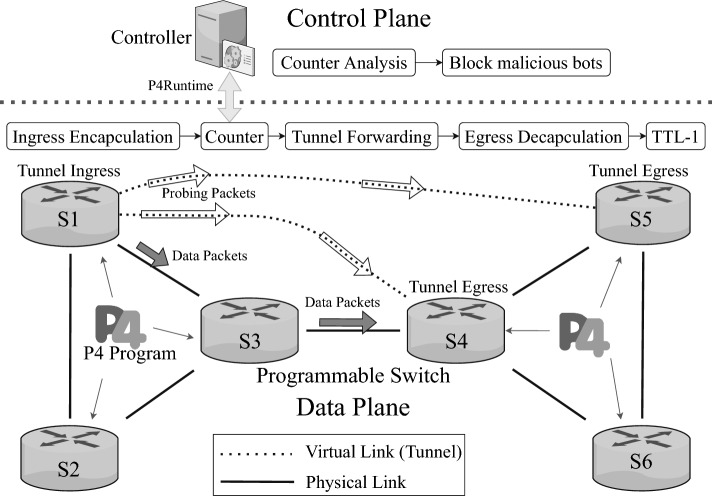
TEPS overview.

Figure 4 illustrates the architecture and workflow of the TEPS system. Hollow arrows passing through dashed lines represent that probing packets are transmitted via virtual tunnels, while solid arrows passing through solid lines represent that normal data packets are transmitted via real physical links. The data plane devices are all programmable switches that support the P4 programming language, allowing each switch to execute the compiled P4 program independently. Normal data traffic entering the network with TEPS implemented is transmitted based on predefined routing algorithms and link paths. In contrast, probing packets are encapsulated at the tunnel’s entrance and forcibly forwarded to the tunnel’s exit. At the tunnel exit, these packets are decapsulated and then transmitted as normal data traffic, with the TTL starting to decrease from this point. In addition, a counter inside the tunnel records the source IP addresses of the transmitted probing packets. This information is analyzed by the controller, which monitors packet transmissions. If the number of probing packets from a specific IP exceeds a predefined threshold within a certain time, the source IP can be identified as a bot and subsequently blocked.

The TEPS processing pipeline is described in detail in Algorithm 1. Upon receiving a packet, the switch first checks its type. If a Traceroute packet is based on TCP, a protocol that prioritizes transmission reliability, or UDP, a protocol that prioritizes transmission speed, the switch applies specialized tunnel-forwarding mechanisms regardless of the protocol used. Although traceroute packets traverse the actual underlying physical links, intermediate devices along the path do not generate responses until the packet reaches its final destination. These intermediate devices do not decrease the TTL value, preventing the host from receiving response messages that disclose each hop’s IP address and RTT. Instead, the TTL value is reduced only when the traceroute packet leaves the tunnel, generating a single response at that point. As a result, the probing host perceives the existence of an additional, seemingly direct path from the edge switch to the destination.

In tunnel mode, ECMP (Equal-Cost Multi-Path)^64^ and LISP (Locator/ID Separation Protocol)^65,66^ play significant roles in mitigating RTT-based topology fingerprinting. The ECMP algorithm uses the CRC (Cyclic Redundancy Check) hash function to determine the forwarding path for packets among multiple equal-cost paths. This process introduces variability in the RTT, which complicates adversaries’ attempts to infer network topology based on timing metrics. On the other hand, LISP separates the IP address space into locators and identifiers, adding an abstraction layer that obscures the actual network layout. By decoupling endpoint identifiers from routing locators, LISP obfuscates the mapping of RTT data to specific network structures. These probing mechanisms increase RTT unpredictability, effectively thwarting adversarial attempts at topology fingerprinting.

In the Anti-RTT-based fingerprinting mechanism, operations on these timing metrics do not affect the normal data packets because only probing packets initiate this mechanism to disrupt their delay, making adversaries elusive and lacking sufficient and effective time evidence to speculate on paths. Therefore, there is no significant end-to-end measurable latency overhead addition for normal data packets. The application of CRC allows for data packet routing based on actual network traffic patterns, making it difficult for adversaries to trace the actual flow of data. By incorporating CRC validation, each packet is appended with a checksum that ensures data integrity during transmission. This not only improves the reliability of data delivery, but also provides a foundation for sophisticated routing strategies. The combination of CRC checksums and topology obfuscation creates a robust defense mechanism against adversaries that attempt to eavesdrop or analyze network traffic. By constantly altering the routes and validating the integrity of each packet, the actual data flow becomes elusive, thwarting attempts to map out or intercept sensitive information. In essence, this strategy leverages the intricacies of modern networking to ensure that the flow of data remains secure and anonymous, making it virtually impossible for adversaries to pinpoint the origin, destination, or exact path of the transmitted information.

Note that an out-of-band control mode is also available for connections between controller and data plane devices. To eliminate the impact of shared links and mitigate the potential risk of APR, we focus on the Traceroute-based probing phase in the in-band mode. For simplicity, TEPS does not incorporate any cipher-related operations.

**Algorithm 1:**
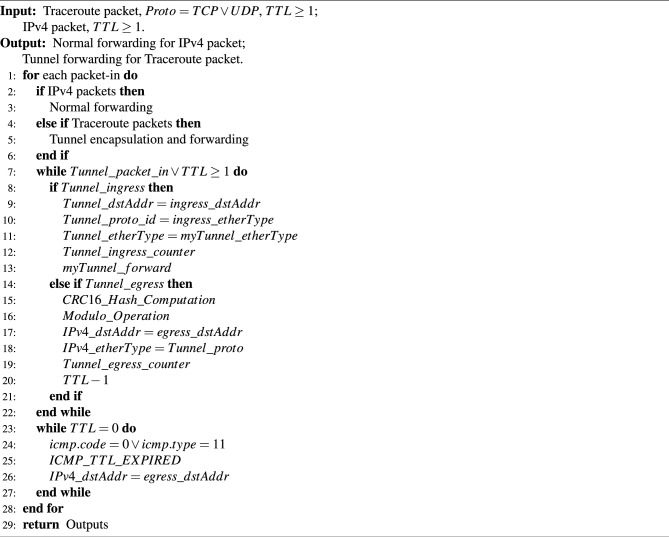
TEPS operation of *Traceroute* packets.

## Evaluation

This section presents the evaluation metrics for assessing the effectiveness of TEPS and provides a detailed analysis of the topology obfuscation results.

**Table 2:** Notation symbol and explanation.

Notation symbol	Explanation
$N\subseteq \{n_1, n_2,..., n_n \}$	Nodes in physical topology
$L\subseteq \textit{N} \times \textit{N}$	Links between physical nodes
$T_{in } \in \{n_1, n_2,..., n_n \}$	Tunnel ingress
$T_{e } \in \{n_1, n_2,..., n_n \}$	Tunnel egress
$\vec {T}\in \textit{T}_{in } \times \textit{T}_{e }$	Tunnel vector from ingress to egress
$P=(N, L)$	Physical topology
$N'\in \{\textit{T}_{in }, \textit{T}_{e } \}$	Nodes in virtual topology
$L'\subseteq \{\vec {T}\}$	Virtual links created by tunnel
$V=(N', L')$	Virtual topology
$F_{l }$	Importance of link
*H*(*P*)	Topology entropy of the topology *P*
$I_{l}$	Importance ratio of link *l*
$F_{avg}(P)$	Importance average of links in topology *P*
$F_{var}(P)$	Importance variance of links in topology *P*
${FR}_{avg}(P)$	Rate of decline of importance average
${FR}_{var}(P)$	Rate of decline of importance variance
$sim(P, V) \in [0, 1]$	Similarity score between topology P and V
*BC*(*n*)	Betweenness Centrality, BC
*CC*(*n*)	Closeness Centrality, CC

### Topology obfuscation effect metrics

We denote our notation symbols and explain the definition with the objectives in Table 2. *N* is the set of all nodes in a physical network topology, which consists solely of P4 programmable switches. $L \subseteq \textit{N} \times \textit{N}$ represents the underlying links in the physical topology. $T_{in }$ and $T_{e }$ refer to the ingress (entrance) and egress (exit) of tunnels established to transmit traceroute probing packets, respectively, and $\vec {T}$ is the tunnel vector. $P=(N, L)$ denotes the real physical topology, consisting of real nodes (switches) *N* and real links *L*, while $V=(N', L')$ represents the virtual topology, which includes virtual links introduced by the tunnel.

An LFA attacker aims to disrupt as many normal users as possible. The importance of a specific link increases with the number of paths that incorporate it, making it a prime target for the attacker. Given that an attacker can detect the path between any ingress and egress nodes, we measure the importance of links based on how frequently they appear on paths between ingress and egress nodes. Assuming that all switch nodes in the topology function as edge switches at the network boundary, all end hosts capable of sending probing packets are connected, allowing each switch to be viewed as the entrance to the tunnel. Although probing packets traverse the invisible underlying physical links, there are actual links hidden under the tunnel, along with the added virtual tunnel links. For any link $l \in \textit{L}$, $F_{l }$ is the frequency of link *l* that appears in all links in *L*. The topology entropy *H*(*P*) can be used to measure the difficulty of identifying critical links in a network. It is calculated as follows:

1 $$\begin{aligned} H(P)= & \sum _{l \in L } I_{l} \cdot {\ln I_{l} }, \end{aligned}$$

2 $$\begin{aligned} I_{l}= & F_l \div \sum _{l \in L } F_l, \end{aligned}$$

$I_{l}$ is the ratio of the importance of the link *l* to the total importance of all links. The topology entropy *H*(*P*) quantifies the security of critical links in the network topology *P*. A lower value of *H*(*P*) indicates a greater discrepancy in the importance of individual links in the network topology *P*, which makes it easier to identify key links. In contrast, a larger *H*(*P*) suggests less discrepancy in link importance, reducing the likelihood of identifying critical links in the network.

TEPS forces traceroute probing packets into tunnel forwarding through virtual links by manipulating the TTL value in the header, while commanding programmable switches to provide silent feedback. This approach hides the bottleneck links as much as possible and reduces their importance. Furthermore, it decreases the frequency of the bottleneck link that appears on the path and prevents them from being visible to adversarial. As a result, the links along all the paths, from the tunnel entrance to the exit, appear equally significant. We use $F_{avg}(P)$ and $F_{var}(P)$ to represent the average and variance of link importance in the topology *P*, respectively. The rates of decline of these metrics after obfuscation are denoted by ${FR}_{avg}(P)$ and ${FR}_{var}(P)$:

3 $$\begin{aligned} {FR}_{avg}(P) = 1- \frac{F_{avg'}(P)}{F_{avg0}(P)}, \end{aligned}$$

4 $$\begin{aligned} {FR}_{var}(P) = 1- \frac{F_{var'}(P)}{F_{var0}(P)}, \end{aligned}$$

$F_{avg0}(P)$ and $F_{avg'}(P)$ refer to the average importance of the links before and after obfuscation, respectively, with the same meaning applied to the variance.

To make the evaluation more intuitive, we compared the similarities between the obfuscated and actual topologies. A common method of measuring similarity between two topologies is Graph Editing Distance (GED), which refers to the sum of the minimum edit operations required to transform a given topology graph into a target topology graph. Specifically, the similarity can be expressed in terms of $similarity \ scores$, denoted as $sim(P, V) \in [0, 1]$:

5 $$\begin{aligned} sim(P, V) = 1- \frac{GED(P, V)}{GED(P, P_0)+GED(V, V_0)}, \end{aligned}$$

*GED*(*P*, *V*) refers to the GED between the physical topology *P* and the virtual topology *V*, while $GED(P, P_0)$ and $GED(V, V_0)$ refer to the GED for constructing a topology graph *P* or *V* from a zero-node tree. These values represent the cost required to remove all elements and create a complete topology. The range of similarity scores *sim*(*P*, *V*) is [0,1], where a higher score indicates greater similarity, and a lower score indicates more differences. As defined by Hou et al. ^19^, the benchmark for the average similarity score between the real network topology and a randomly generated topology is 0.6. The closer the obfuscation solution is to this value, the more effective it can be considered.

*BC*(*n*) (Betweenness Centrality) and *CC*(*n*) (Closeness Centrality) are static indicators used to determine the importance of an individual note in the topology, as defined by Kim et al.^18^.

6 $$\begin{aligned} & BC(n) = \sum _{a\ne b\in N} \frac{L_{ab}(n)}{L_{ab}}, \end{aligned}$$

7 $$\begin{aligned} & CC(n) = 1 {\div } \sum _{a\ne b\in N}L_{s\longrightarrow n}, \end{aligned}$$

$L_{ab}$ refers to the reachable paths between nodes *a* and *b*, while $L_{ab}(n)$ represents the paths that pass through node *n*. $L_{s\longrightarrow n}$ denotes the shortest distance from any node *s* to node *n*. The higher the values of BC and CC for a node, the more important that node is in the network. Considering research^67^, which demonstrates that traceroute packets directed toward near nodes with high BC and CC values are highly suspicious, these metrics provide an effective means of evaluating the effectiveness of defense methods.

**Table 3:** Topology information.

Name of topology	Compuserve	Gambia	SwitchL3
Number of nodes	14	28	42
Number of links	17	28	63

**Fig. 5:**
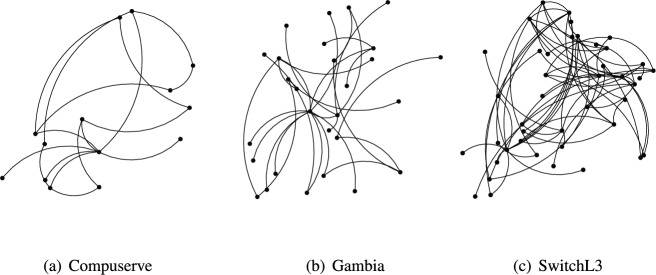
Topology structure.

### Testbed setup and prototype

Our experimental environment is based on Ubuntu 20.04 LTS. TEPS is implemented using P4 ^45^ on the target software switch *simple_switch_grpc* ^68^, with its syntax conforming to the *v1model* architecture ^69^. We employ the ONOS controller ^70^, which supports both P4 and P4Runtime ^43^, as the control-plane API to interface with the data plane. The network topology is constructed using Mininet ^71^, and the environment is configured with Python 3.8, Protobuf 24.0, and gRPC 1.3.2. We used three real-world network topologies of varying sizes, available on *Topology Zoo*^72^, with details shown in Table 3 and Fig. 5.

**Fig. 6:**
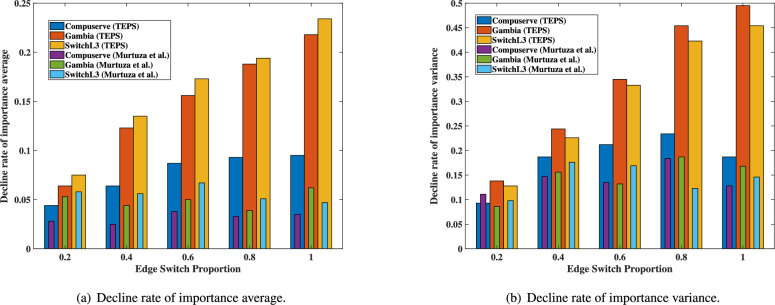
Comparison of decline rates of importance metrics.

We assume that each node in the topology can function as an edge switch with at least one end host connected. While Traceroute enables adversaries to determine the hop count along the path to the destination, TEPS provides only a direct mapping from the edge switch to the destination. Adversaries send traceroute probing packets from compromised end hosts located at various points on the network to a desired destination IP address. We conducted experiments on these three topologies to investigate the relationship between the portion of nodes designated as edge switches and the indicators mentioned above.

### Result and effectiveness

Murtuza et al.^24^ proposed a defense method to disrupt topology mapping by randomly reducing the TTL value by an integer between 0 and 3. This approach introduced randomness in the concealment or addition of nodes within the topology. In our evaluation, we compare TEPS with their method using various quantitative metrics to assess performance and effectiveness.

**Fig. 7:**
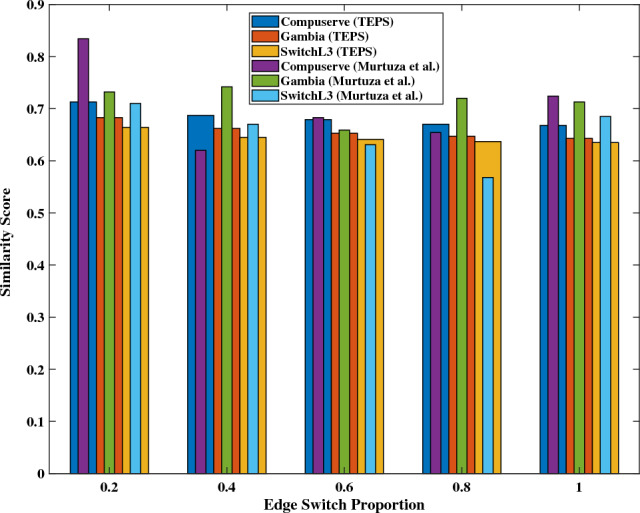
Similarity score between the virtual topology and the real topology.

**Fig. 8:**
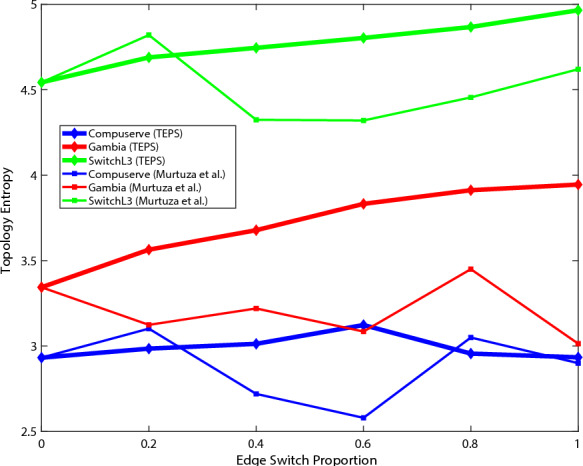
Change of topology entropy.

**Table 4:** Comparison of Metrics between TEPS and Murtuza et al. methodologies.

Metrics	TEPS	Murtuza et al.
**Decline rate of importance average**		
–Compuserve	0.05~0.08	0.02~0.04
–Gambia	0.06~0.22	0.05~0.06
–SwitchL3	0.07~0.23	0.05~0.07
**Decline rate of importance variance**		
–Compuserve	0.09~0.22	0.11~0.17
–Gambia	0.14~0.49	0.09~0.18
–SwitchL3	0.13~0.45	0.10~0.17
**Similarity Scores**		
–Compuserve	0.68~0.71	0.63~0.83
–Gambia	0.67~0.68	0.68~0.74
–SwitchL3	0.65~0.66	0.57~0.71
**Topology Entropy**		
–Compuserve	2.9~3.2	2.6~3.2
–Gambia	3.3~3.9	3.3~3.3
–SwitchL3	4.5~4.9	4.3~4.7

Fig. 6 illustrates the rate of decline in the average and variance of the importance of the link across the three topologies. Regardless of the proportion of edge switches, the TEPS shows a significantly higher rate of decline in importance. The decline reaches approximately 0.25 for the importance average, while it drops by as much as 0.5 for the importance variance. Furthermore, the decline rates show a consistent upward trend as the proportion of edge switches increases, except in the small-scale network topology *Compuserve*, where the decline rate in variance decreases again when the proportion of edge switches reaches 1. In contrast, Murtuza et al. method achieves only 0.07 and 0.2 reductions in importance average and variance, respectively, and does not exhibit any discernible pattern based on the edge switch proportion.

**Fig. 9:**
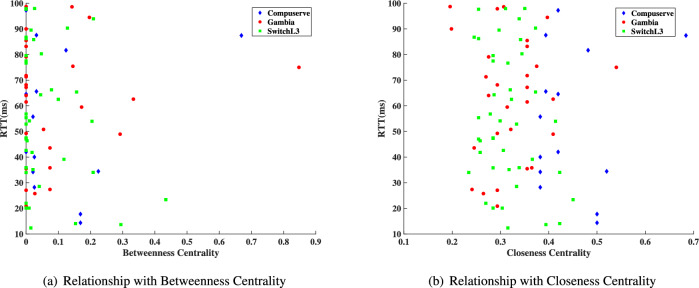
Distribution of RTT values for each node.

Fig. 7 illustrates the similarity score between the inferred topology and the real topology by edge-switch proportion. For all types of inferred topologies, the similarity score approaches 0.6 as the TEPS deployment increases from partial to complete. This result indicates that adversaries are unable to gather sufficient evidence to reconstruct the real topology and instead obtain a random inferred topology. In contrast, the similarity score for the Murtuza et al. method generally remains significantly higher than that for TEPS. Although there are cases where it exhibits lower similarity scores, the distribution appears irregular.

Modifying the portion of edge switches will increase the importance of virtual links while decreasing the importance of critical links in real networks. Thus, it is crucial not only to reduce the importance differences between links, but also to increase the structural entropy of the topology. Fig. 8 shows the change in the topology entropy. TEPS shows a gradual increase in the topology entropy as higher levels of obfuscation are applied. However, in the case of *Compuserve*, the entropy peaks at a proportion of 0.6 and then shows a decreasing trend thereafter. These experimental results indicate that, in small-scale networks, a higher application of TEPS technology increases the significance of virtual links compared to the underlying real links. This results in greater differences in link importance, leading to a decrease in topological entropy and a decrease in the rate of importance. However, based on the results from most topologies, which show that the application of TEPS makes it increasingly challenging to infer the real topology, TEPS remains effective in obscuring the topology.

**Fig. 10:**
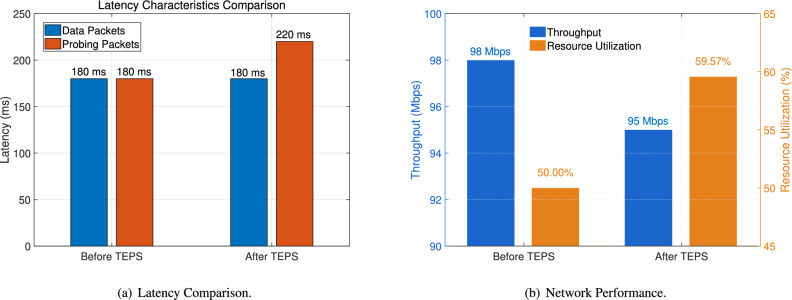
Computational overhead.

**Fig. 11:**
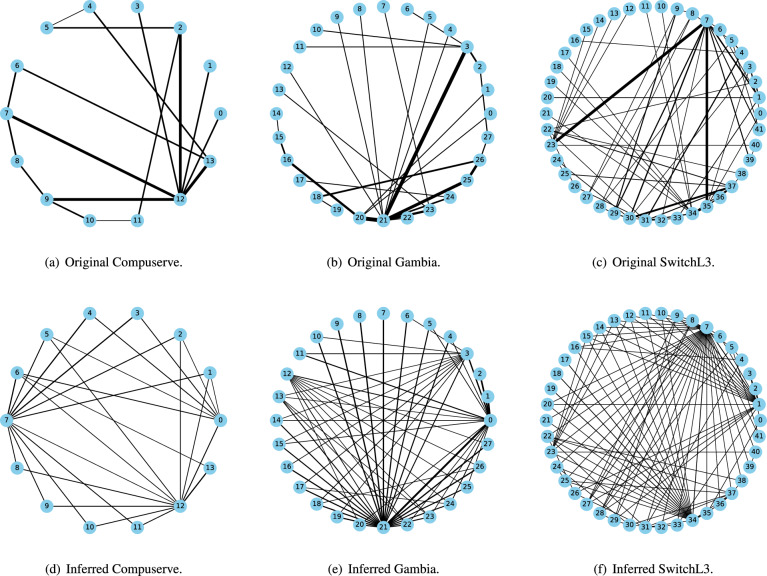
Original topology versus inferred topology. The importance of each link is expressed by the thickness of the line.

Table 4 presents the numerical ranges of the values used in the figures comparing Murtuza et al.’s approach with TEPS. Overall, TEPS shows a higher decline rate in link importance and greater topology entropy, indicating more effective disruption of adversarial inference. While Murtuza et al.’s method occasionally yields a lower similarity score, TEPS consistently maintains a narrower range of similarity scores, suggesting more stable and reliable obfuscation. These numerical results align with the visual observations from the figures and further demonstrate the effectiveness of TEPS.

Due to invisibility in the tunnel, TEPS manipulates the RTT of the packets secretly. Fig. 9 illustrates the RTT values based on the BC and CC values. Typically, nodes with high BC and CC values in a network tend to exhibit high RTT averages when measured from other nodes in the network. This RTT value increases linearly with the complexity of the node. Additionally, due to the external invisibility of the tunnel in TEPS, adversaries are unable to detect the paths of probing packets. As a result, performing network tomography by analyzing the RTT values becomes difficult, even when those values are similar to the true RTT. Also, various factors, such as asymmetric feedback, forwarding paths, internal routing protocols, and loopbacks, can also exist within the tunnel. These factors depend on both static and dynamic computations of the links and nodes, which significantly enhance the randomness of the RTT. TEPS can effectively disrupt these patterns, strengthening its resilience against RTT-based fingerprinting techniques.

To assess TEPS’s impact on legitimate traffic, we quantify routing deviations and end-to-end delay distributions across diverse scenarios. As shown in Fig. 10, it demonstrates that TEPS lightweight operation in software-based P4 environments, throughput shows marginal decrease (98→95 Mbps, <3% dropped) and resource utilization highlights computational overhead 15-20% increased. This contrast validates TEPS’s lightweight nature–maintaining network performance while introducing controlled resource costs under operational constraints. Additionally, there was no significant negative impact on the average latency of normal data packets. While this aligns with software-level SDN approaches like Murtuza et al.’s, hardware P4 switches inherently outperform traditional SDN devices in scalability and overhead. For instance, P4-programmable ASICs (e.g., ©Intel$^\circledR$ Tofino$^\text {TM}$ switches^73^) achieve line-rate forwarding at 6.5 Tbps with sub-microsecond latency (Bosshart et al., 2014^40^), whereas conventional SDN switches incur 10–100× higher processing delays for dynamic policies. This architectural advantage positions TEPS for efficient deployment in large-scale networks without compromising throughput. These results underscore TEPS’s ability to harmonize security enforcement with QoS guarantees by leveraging lightweight path adjustments and predictive load balancing. The controlled trade-offs align with ISP operational priorities. Based on the results, the marginal throughput reduction (3%) and increased resource utilization (15–20%) under TEPS primarily stem from its security-driven packet processing. TEPS introduces lightweight in-network checks to distinguish probing packets from legitimate traffic, requiring additional header inspections and path validation. This verification process, coupled with predictive load-balancing computations for rerouting suspicious flows, incurs minimal but measurable computational overhead. However, P4’s programmability optimizes these operations via parallelized pipelines, ensuring most forwarding remains at line rate. The trade-off is intentional: TEPS prioritizes attack mitigation with controlled resource costs while preserving QoS for legitimate traffic. Hardware P4 switches (e.g., Tofino^73^) further minimize overhead via ASIC acceleration, making such trade-offs negligible in practice.

Fig. 11 illustrates the original topologies and the topologies inferred by the adversaries. Since TEPS conceals intermediate information and diminishes the importance of each link, making it difficult for adversaries to infer an accurate topology. Important links remain undetected or are overwritten by newly added virtual links, and even if they are detected, they are assigned much less importance. Consequently, TEPS’s obfuscation increases the complexity and uncertainty of the topologies, weakening adversaries’ ability and willingness to analyze the inferred topology and carry out subsequent attacks.

## Discussion

In this section, we discuss additional functions and potential limitations of TEPS. First, TEPS is capable of detecting adversaries attempting malicious probing. Legitimate probe packets are typically distributed uniformly across hop counts, whereas malicious packets tend to concentrate at specific hop values ^67^. To detect such behavior and locate adversaries, TEPS places counters at the entrance and exit of each tunnel to record the number of probing packets sent to and received from each node. These statistics are then transmitted to the controller via the P4Runtime API ^43^. By analyzing this data, the controller can identify and isolate hosts that generate abnormal volumes of probing packets, revealing potential reconnaissance attempts.

Proactive defense operates on the principle of strategic cost imposition rather than absolute prevention. By dynamically obfuscating the attack surface, it compels adversaries to expend disproportionate resources on reconnaissance, analysis, and adaptation. Rational attackers, guided by cost-benefit considerations, are deterred when the effort required to penetrate defenses outweighs the potential gains. While persistent adversaries may eventually infer partial aspects of the system, extended time-to-compromise (e.g., months of sustained probing) and recurring adaptation cycles (e.g., tunnel reconfiguration) render such attacks economically unsustainable. This approach aligns with the “moving target” doctrine in cybersecurity, in which defenders leverage asymmetries in adaptation costs to erode attacker persistence and redirect their efforts toward alternative, softer targets ^22^. As a result, TEPS contributes to long-term system robustness and resilience.

Although TEPS effectively isolates probing-specific obfuscation from legitimate traffic, distinguishing benign diagnostic tools (e.g., traceroute) from adversarial reconnaissance remains a non-trivial challenge. Sophisticated attackers may mimic legitimate probing behaviors (e.g., incremental TTL scans) to evade detection. While adaptive thresholds are used to differentiate traffic patterns, they cannot fully infer user intent without contextual awareness (e.g., authentication, service dependencies). This limitation is inherent in packet-level feature-based defenses and motivates future work in protocol fingerprinting and machine learning-assisted intent inference. To mitigate operational risks, TEPS can be complemented with administrative policies, such as whitelisting authorized diagnostic IP addresses.

Many proactive defenses that reroute Traceroute packets can lead to unintentional service unavailability for legitimate users^15,22,23^. This typically results from generating fake topologies in advance, which may reroute or delay normal traffic and increase overall system load^14,16,17^. Moreover, identifying malicious users heavily depends on the accuracy of classification algorithms. Misclassifications may lead to false positives or negatives, compromising the reliability of the defense and disrupting legitimate network diagnostics ^19^. TEPS, in contrast, actively obfuscates topology only for probing packets. Issues related to legitimate diagnostics can be mitigated by enforcing centralized authorization policies or restricting access to trusted users. However, automated differentiation between malicious and legitimate Traceroute users remains an open challenge requiring further research.

While TEPS demonstrates strong performance in P4 software switches ^68^ using the v1model architecture^69^, transitioning to hardware platforms (e.g., Intel^®^ Tofino^TM^ switches^73^) introduces several challenges. Hardware resource constraints, such as limited TCAM memory and fixed-function pipeline stages, may restrict the frequency of topology mutations, and hardware-induced latency may impact the responsiveness of threshold-triggered adaptations. These limitations can be partially addressed through optimized techniques. For example, excessive virtual path updates may exhaust memory if the size of the state table exceeds hardware capacity. Therefore, system compatibility and scalability must be evaluated based on the specific deployment environment. Additionally, packet processing delays introduced by hardware pipelines may limit responsiveness. Notably, 68% of the optimizations in TEPS stem from existing hardware-aware obfuscation techniques, indicating that current hardware capabilities already support substantial defenses. Future formalization efforts will aim to propose novel methodologies while also systematizing these empirically validated solutions.

Any operation that modifies or encapsulates packet headers may cause a loss of fine-grained information, such as the precise location of packet loss. This can be addressed by extending TEPS with supplementary functionality through a centralized controller. Furthermore, while TEPS assumes a deployment model in which traditional routers are replaced with programmable switches, full-scale upgrades may not be feasible in large networks due to cost constraints. In such scenarios, partial deployment becomes a viable option, particularly given that our evaluation demonstrates topology obfuscation improvements even in partially deployed environments.

The integration of SDN and P4 for active defense has been validated in both terrestrial and satellite networks. A representative example is the CONECT-PES project ^74^, which integrates SDN controllers, Mininet, and P4-programmable switches to manage traffic within LEO satellite constellations. This architecture achieved dynamic QoS enforcement using GNU Radio for signal processing and P4 for packet forwarding logic, demonstrating scalability under varying network loads. For LFA mitigation, P4’s hardware compatibility ensures deployability, and modern switches such as Intel Tofino support up to 32K flow-table entries, which is sufficient for mid-sized network obfuscation. Additionally, future work from the CONECT-PES project proposes FPGA-based P4 implementations to further improve throughput. These cases confirm that our virtualized experiments reflect real-world constraints and align with ongoing efforts to integrate large-scale SDN implementations, such as Huawei’s NCE controller. They also reinforce the practicality and scalability of our topology obfuscation framework. TEPS can thus be deployed in real-world networks equipped with P4 switches to proactively mitigate malicious probing and prevent subsequent network attacks.

## Conclusion and future work

This paper presented TEPS, a proactive defense system designed to counteract adversarial reconnaissance. Implemented in a P4-based SDN with flexible programmable switches, TEPS leveraged the concept of tunneling for secure transmission, forwarding traceroute probing packets through a specialized tunnel to obstruct adversary reconnaissance. Experimental results demonstrated that TEPS significantly achieved topology obfuscation in realistic network topologies, strengthening resistance against RTT-based fingerprinting and enhancing the security of the SDN system.

For future work, we plan to integrate advanced AI technologies to enhance TEPS by enabling automated tunneling of packets suspected to be malicious, extending its capabilities beyond Traceroute-based detection to proactively counter a broader range of attacks beyond LFAs. Additionally, we aim to leverage state-of-the-art AI models for real-time attack detection in SDN, allowing programmable switches to identify and mitigate emerging threats autonomously. By incorporating these AI-driven capabilities, TEPS can significantly improve the accuracy of malicious packet identification and the adaptability of SDN defenses, further strengthening network security against evolving cyber threats.

## Data Availability

All data generated and/or analyzed during this study are included in this published article. Detailed information about the network topologies used in this study can be accessed from the Internet Topology Zoo (https://github.com/sk2/topologyzoo).
